# Supplementary material for: Effects of Different Storage Temperatures on Bacterial Communities and Functional Potential in Pork Meat
Source: Foods. 2022 Aug 2;11(15):2307. doi: 10.3390/foods11152307 (PMC9367820; doi:10.3390/foods11152307)
Supplement: Supplementary file 1 [file foods-11-02307-s001.zip › foods-1830967-supplementary.pdf]

Supplementary files

Effects of different storage temperatures on bacterial communities and functional potential in pork meat

Fan Zhao [2], Zhenqian Wei [1], Guanghong Zhou [1], Karsten Kristiansen [2,3,4,*] and Chong Wang [1,2,*]

[1] College of Food Science and Technology, Nanjing Agricultural University; Key Laboratory of Meat Products Processing, Ministry of Agriculture; Jiangsu Collaborative Innovation Center of Meat Production and Pro-cessing, Quality and Safety Control, Nanjing, 210095, P.R. China; 2019108080@njau.edu.cn (Z. W.); guanghong.zhou@hotmail.com (G. Z.)

[2] Laboratory of Genomics and Molecular Biomedicine, Department of Biology, University of Copenhagen, Copenhagen 2100, Denmark; zhao.fan@bio.ku.dk

[3] BGI-Shenzhen, Shenzhen 518083, P. R. China

[4] Institute of Metagenomics, Qingdao-Europe Advanced Institute for Life Sciences, BGI-Qingdao, Qingdao 166555, P. R. China

* Correspondence: kk@bio.ku.dk (K.K); chongwang@njau.edu.cn (C.W)

Supplementary Materials and methods

Protein degradation

Three grams of pork meat stored at different temperature at different time points were used to extract total protein using the RIPA lysis buffer system (YEASEN, Shanghai, China) with supplement of protease inhibitor cocktails (Roche, Basel, Switzerland). The concentration of extracted protein in each sample was determined using a BCA protein assay kit (Thermo Fisher Scientific, Waltham, MA, USA) and was adjusted to a final concentration of 5 mg/mL. Samples were then mixed with 4x loading buffer (GenScript, NJ, USA) and were heated in water bath at 95 ℃ for 10 min. A total of 30 μg proteins of each sample were loaded on a 4-12% sodium dodecyl sulfate-polyacrylamide gel electrophoresis gradient gel (GenScript, NJ, USA). The gels were first run at 80 V for 20 min, and then at 120 V for 80 min. Gels were stained by Feto SDS-PAGE staining buffer (Absci, Vancouver, WA, USA) for 30 mins, followed by de-staining and scanning using an image analyzer (GE Healthcare, Chicago, IL, USA).

TCA-soluble peptides

The pork meat samples were prepared according to previous published method with a slight modification [1]. Briefly, two grams of the meat sample was added into 18 mL pre-cooled 5% (w/v) TCA solution. Then, the mixture was homogenized using a homogenizer at the speed of 19,000 r/min for 1 min. The homogenate was kept standing on ice for 1 hour, and then centrifuged at 12,000g for 15 min at 4 ℃. Finally, the supernatant was transferred to a new tube and the pellet was discarded. The measurement of TCA-soluble peptides was performed according to method of Lowry et al [2], the result was presented as μmol tyrosine/g meat.

Supplementary Figure S1

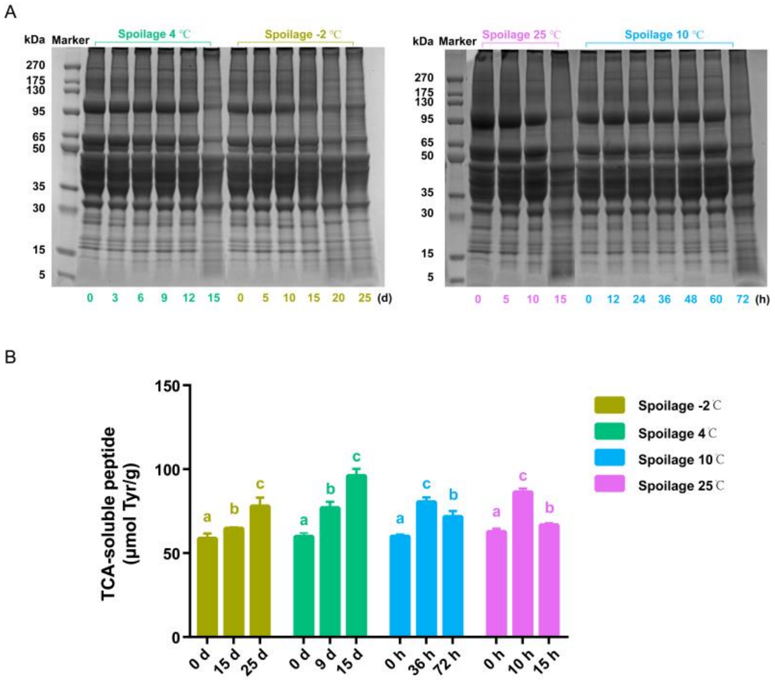

Supplementary Figure S1. Protein degradation of pork meat during storage under different temperatures. (A) SDS-PAGE of pork meat protein under storage at -2, 4, 10, and 25 °C. (B) TCA-soluble peptide in pork meat protein under storage at -2, 4, 10, and 25 °C. Fresh to semi-fresh stage: from 0d to 15d for storage under -2 ℃; from 0d to 9d for storage under 4 ℃; from 0h to 36h for storage under 10 ℃; from 0h to 10h for storage under 25 ℃. Semi-fresh to spoiled stage: from 15d to 25d for storage under -2 ℃; from 9d to 15d for storage under 4 ℃; from 36h to 72h for storage under 10 ℃; from 10h to 15h for storage under 25 ℃. Values are shown as mean±SD, a, b, c represent significant difference ($P < 0.05$) between different stages under each storage temperature analyzed by one-way ANOVA.

References

1. Benjakul, S.; Visessanguan, W.; Tueksuban, J.; Tanaka, M. Effect of some protein additives on proteolysis and gel-forming ability of lizardfish (Saurida tumbil). *Food Hydrocolloids* **2004**, *18*, 395-401.
2. Lowry, O.H.; Rosebrough, N.J.; Farr, A.L., Randall, R.J. Protein measurement with the Folin phenol reagent. *Journal of Biological Chemistry* **1951**, *193*, 256-275.
